# Hormonal Control of Lateral Root and Nodule Development in Legumes

**DOI:** 10.3390/plants4030523

**Published:** 2015-08-07

**Authors:** Sandra Bensmihen

**Affiliations:** 1 INRA, Laboratoire des Interactions Plantes-Microorganismes (LIPM), UMR441, F-31326 Castanet-Tolosan, France; E-Mail: Sandra.bensmihen@toulouse.inra.fr; Tel.: +33-561-285463; Fax: +33-561-285061; 2 CNRS, Laboratoire des Interactions Plantes-Microorganismes (LIPM), UMR2594, F-31326 Castanet-Tolosan, France

**Keywords:** auxin, peptides, lateral root, local/systemic regulation, miRNA, *Medicago truncatula*

## Abstract

Many plants can establish symbioses with nitrogen-fixing bacteria, some of which lead to nodulation, including legumes. Indeed, in the rhizobium/legume symbiosis, new root organs, called nodules, are formed by the plant in order to host the rhizobia in protective conditions, optimized for nitrogen fixation. In this way, these plants can benefit from the reduction of atmospheric dinitrogen into ammonia by the hosted bacteria, and in exchange the plant provides the rhizobia with a carbon source. Since this symbiosis is costly for the plant it is highly regulated. Both legume nodule and lateral root organogenesis involve divisions of the root inner tissues, and both developmental programs are tightly controlled by plant hormones. In fact, most of the major plant hormones, such as auxin, cytokinins, abscisic acid, and strigolactones, control both lateral root formation and nodule organogenesis, but often in an opposite manner. This suggests that the sensitivity of legume plants to some phytohormones could be linked to the antagonism that exists between the processes of nodulation and lateral root formation. Here, we will review the implication of some major phytohormones in lateral root formation in legumes, compare them with their roles in nodulation, and discuss specificities and divergences from non-legume eudicot plants such as *Arabidopsis thaliana*.

## 1. Legume Plants and Root Development

Nitrogen is indispensable for life and is required in many biochemical processes such as nucleic acid and amino acid biosynthesis. Despite the huge quantity of dinitrogen (N_2_) present in the air, mineral forms of nitrogen (nitrate and ammonium) are often limiting in the soil. Although four different orders of flowering plants (Rosales/Fagales/Cucurbitales/Fabales) are able to associate with micro-organisms that fix dinitrogen, the Leguminosae has the highest proportion of species able to nodulate [1]. This nodulation capability is thought to have favored the success and radiation of the Leguminosae. Indeed, the Leguminosae (or Fabaceae) is the third largest family of flowering plants and most of its 20,000 species are able to nodulate. Nodulation implies the formation of a new organ, the nodule, that hosts soil bacteria, called rhizobia, in conditions optimal for nitrogen fixation. To some extent, nodules resemble lateral roots and they originate from the same root cell layers. Two types of nodules can be discriminated depending on the persistence or not of their meristematic activity. Hence, most temperate legumes, like *Medicago* ssp., *Pisum sativum* (pea) and *Trifolium* sp. (clover), produce indeterminate nodules whose meristems persist over the entire nodule lifespan while other legumes, like *Lotus japonicus* (Lotus) or *Glycine max* (soybean), produce determinate nodules, whose meristematic activity stops early on during nodule development giving rise to small, round shaped nodules. In legume species, both the onset of nodulation and rhizobial entry into the root are dependent on a specific molecular dialog involving the production by the rhizobia of Nodulation Factors (NF) which also control notably host specificity [2]. Downstream of NF perception, signal transduction notably involves calcium spiking ([3] for review).

Hormones are regulatory signals produced within an organism and usually active at very low concentrations and at a distance from their site of production. Plants, like other eukaryotes, produce different types of hormones. In contrast to animals, it is more difficult to specify which cells produce phytohormones and distinguish them from cells that perceive the hormone signals. However, hormonal control of plant growth is well established and was first studied with auxin at the beginning of the 20th century [4]. In Leguminosae, many phytohormones control both root and nodule development. Interestingly, Liang and Harris [5] have studied the ability of various legumes and non-legumes plants to respond to the phytohormone abscisic acid (ABA) by increasing their lateral root (LR) density and hypothesized that the common predisposition of plants to form nodules may be linked to a difference in ABA sensitivity. Hence Leguminosae may have evolved specific hormonal sensitivities that enable them to control two types of “lateral” root organs, *i.e.*, LR and nodules.

In this review, we will concentrate on nodulating legumes from the Papilionoideae clade that have been more widely studied and especially on *Medicago truncatula* (a diploid model close to alfalfa, *M. sativa*), soybean (*Glycine max*), Lotus (*Lotus japonicus*), pea (*Pisum sativum*) and clover (*Trifolium repens*). We will review the ability of endogenous and exogenous signals to modulate the root development of these plants and show how this modulation is linked to hormonal control. When relevant, we will highlight the dual hormonal controls on root and nodule development and possible differences between hormonal control of root development in legumes and *Arabidopsis thaliana* (that does not nodulate), as summarized in Table 1.

**Table 1:** Summary of the action of the main phytohormones on root development and nodulation in legumes and root development in Arabidopsis or other non-legume dicot species (when specified).

Hormone	Action on Root Development in Legumes	Action on Nodulation	Action on Root Development in Arabidopsis or Other Dicots	References
**Auxin**	+ on LRF at low doses, − at higher doses	+ at low doses on indeterminate nodules	+ on LRF at low doses, − at higher doses	[6,7,8,9]
**Cytokinins**	− on LRF	+ on nodulation	− on LRF	[10,11,12]
**Abscisic acid (ABA)**	+ on LRF at low doses (≤ 10^−7^ M), − at higher doses + on intermediate LRF stages and meristematic activity	− on nodule development and NF signaling	− on LRF at 10^−7^ M − on LRP emergence and meristematic activity	[5,13,14,15,16]
**Ethylene**	+ on LRF at low doses, − at higher doses. Inhibits primary root length	−	− on LRF and primary root growth through auxin interaction	[17,18,19,20,21]
**Nitric oxide**	Present in LRP. Necessary downstream of auxin for LRF?	Necessary for early infection and nodule primordia formation. Accelerates nodule senescence	+ on LRF through reactivation of cell cycle genes in tomato, downstream of auxin. + on LRF in sunflower seedlings	[8,22,23,24,25,26,27]
**Jasmonic acid**	+ on LRF, − on primary root length	− on nodulation by acting on NF signaling	+ on LRF and − on primary root length	[28,29]
**Brassinosteroids**	− on primary root length and LRF	± (*cf.* [30], for review)	Promote LR emergence at low doses (10^−8^ M) Promote LRF and root apical meristem maintenance	[31,32]
**Gibberelic acid**	Pea biosynthetic mutants are dwarf with fewer LR	May require an optimum concentration + on pea, − on lotus [33]	Inhibits LRF (in poplar)	[31,34]
**Strigolactones**	Reduce LRF from 10^−7^ M	+ at low doses in *M. truncatula* and in pea, lotus; − at higher doses	− on LRF (phosphate dependent conditions)	[35,36,37,38]
**CLE/CLV1**	− on LRF (dependent on nitrate status in the shoot)	− on nodulation through the AON pathway	− on LRF in nitrate limiting conditions	[39,40,41,42]
**CEP/LRR-RLK**	Reduces LRF	Enhances nodulation in a systemic pathway	Reduces LRF in nitrate limiting conditions (systemic action)	[43,44,45]

Data is compiled for legumes from literature in *M. truncatula*, *L. japonicus*, *Glycine max* (soybean) and *Pisum sativum* (pea). LRF: Lateral Root Formation. CLE: CLAVATA3/EMBRYO-SURROUNDING REGION peptides; CLV1: Clavata 1; CEP: C-terminally Encoded Peptide; LRR-RLK: Leucine Rich Repeat Receptor Like Kinase.

## 2. Root Anatomy: Cell Layers and Definitions

The root system plays a major role in plants as it ensures the anchorage of plants in the soil and their nutrition. Roots are also highly reactive to exogenous “signals” such as nutrient availability or biotic stresses (pathogens or symbionts) and their development is, therefore, highly plastic. The root system of eudicot species is classically composed of a primary root (which directly originates from the radicle formed in the embryo) and LR that are formed post embryonically and are thus very responsive to environmental cues [46]. When talking about root development, one generally takes into account both the primary root growth and LR formation (LRF), these two parameters can be recapitulated by the LR density (number of LR per cm of primary root) [47].

**Figure 1:**
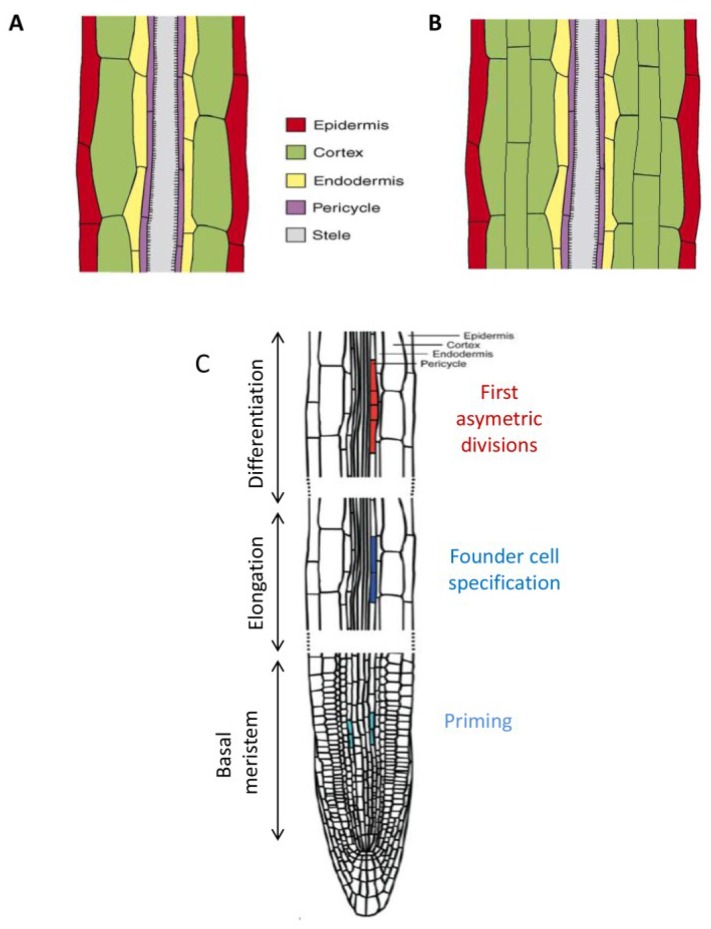
Schematic organization of the primary root. (**A**) The different cell layers in a longitudinal section along the primary root in Arabidopsis, note that there is only one cell per root cell layer; (**B**) the different cell layers in a longitudinal section along the primary root of *M. truncatula*, showing several cortical cells (only three are represented whereas four to five layers are usually observed); (**C**) Longitudinal organization of the primary root in the younger region of the root (close to the root tip), showing the different zones of the LR specification steps. (**A**) and (**B**) are adapted from [50]; (**C**) is adapted from [49].

The primary root of Angiosperms can be divided into different developmental zones along the proximo-distal axis, following a differentiation gradient [48]. Hence, close to the root tip is the root apical meristem, whose cells are dividing and will give rise to the different cell layers of the root. Above the root tip is the elongation zone, where the cells stop dividing and start elongating. In between these two zones is the basal meristem, which has a critical role in specifying LR founder cells [49]. Above the elongation zone (towards the shoot) is the differentiation zone where the cells differentiate, for example to form root hairs (*cf.* Figure 1C).

On the radial axis, the root is formed of several concentric cell layers (*cf.* Figure 1A,B). The innermost layer of the root is named the stele and comprises the vessels (xylem and phloem) that are responsible for the movement of water and nutrients inside the plant. The stele is surrounded by the pericycle, a tissue that is notably able to dedifferentiate to initiate the formation of new LR. Next is the endodermis, that is generally suberified (in the older part of the root) and forms a natural barrier between the inner part of the root and the cortex. The root cortex is generally composed of several cell layers, although Arabidopsis has only one cell layer for each of these root tissues (*cf.* Figure 1A,B). The outermost cell layer is the root epidermis (or rhizodermis) whose cells can form root hairs that are privileged sites of water and nutrient uptake.

## 3. Auxin, a Major Regulator of LRF and Nodule Development

Molecular root development studies have mostly been conducted on the model species *Arabidopsis thaliana*. This plant is a small dicot species from the Brassicaceae family which has only a single cell per root cell layer (Figure 1A). Thanks in particular to its simple root architecture, Arabidopsis has been a very powerful model to unravel the early steps of LRF and the role of the phytohormone auxin in this process.

Hence, it has been shown that LR originate from divisions in the pericycle. However, LR are formed sequentially and not all the cells from the pericycle are competent to form LR ([51,52] and references therein). Indeed, it was shown that only some of these pericycle cells are “primed” to become LR founder cells. In Arabidopsis, these LR founder cells are always found opposite a protoxylem pole and are characterized by an increased auxin accumulation [53]. Moreover, De Smet and collaborators have shown that priming is linked to a pulsatile auxin response in protoxylem cells in the basal meristem of the root [54]. Following the priming event, founder cell specification occurs within a developmental window that is located in a well-defined zone along the primary root axis where auxin content and response are minimal [55].

**Figure 2:**
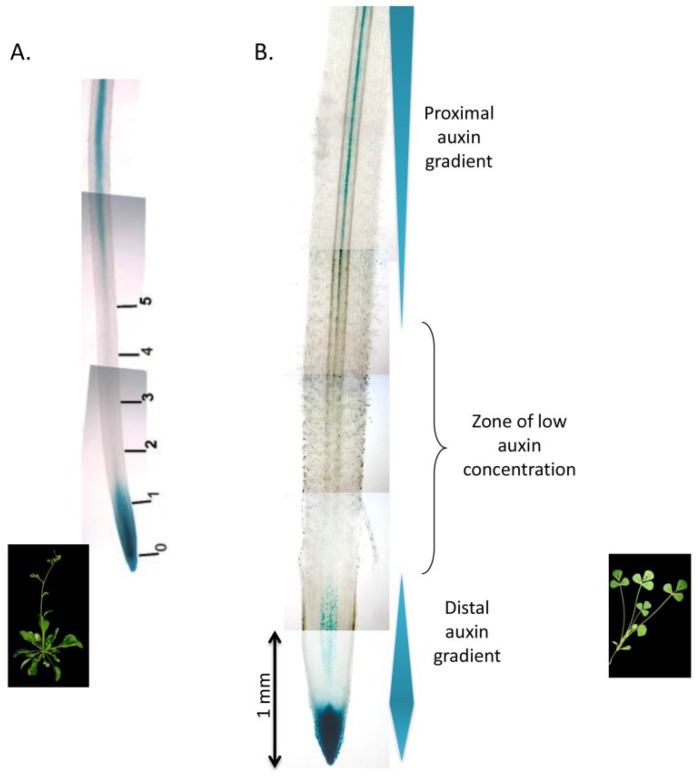
DR5:GUS reporter gene activity reflecting auxin gradients in *M. truncatula* and *A. thaliana* primary roots. (**A**) DR5:GUS expression gradient in Arabidopsis, as described in [55]; (**B**) DR5:GUS expression gradient in *M. truncatula* [63]. GUS activity appears in blue.

Auxin is the major phytohormone governing LRF and primary root growth in all plants so far studied, although most of the molecular evidence has been shown on Arabidopsis. For instance, it has been shown that local auxin accumulation in pericycle cells is sufficient to trigger LRF in Arabidopsis [53]. Auxin is also involved at later steps of LRF, such as the preparation of LR emergence from the main root [56]. Auxin is mainly synthesized in the shoot, but a portion is also locally produced in the root [57]. Auxin is thus moving from the shoot to the root (in an acropetal movement) and from the root to the shoot (mainly recycled at the root tip via auxin efflux transporters) in a basipetal movement [58]. Hence, two auxin gradients are formed in the root (*cf.* Figure 2). Auxin perception can be monitored by a reporter system, consisting of the auxin sensitive synthetic promoter DR5 fused to a GUS or GFP reporter gene. DR5 is a sevenfold tandem repeat of auxin responsive elements originally found in an auxin responsive *GH3* gene from soybean [59]. Thus, DR5 reporters are suitable for legumes and have been used to assess auxin perception during root and nodule development [60,61]. As shown in Figure 2, *M. truncatula* displays the same auxin gradients as Arabidopsis as revealed by the DR5:GUS promoter, and notably the same zone of auxin minimum above the basal meristem. We have also shown that the LR initiation zone is located at the beginning of the differentiation zone in *M. truncatula* [61] as shown in Arabidopsis [62].

Using the same DR5:GUS transgenic plants, we have described zones of auxin perception during LRF in *M. truncatula* [61]. We have thus shown that, as in Arabidopsis, DR5:GUS activity precedes the first divisions of pericycle cells. Auxin perception also precedes and possibly triggers the divisions of other cell layers contributing to LRF in *M. truncatula*, namely the endodermis and inner cortex [61].

**Figure 3:**
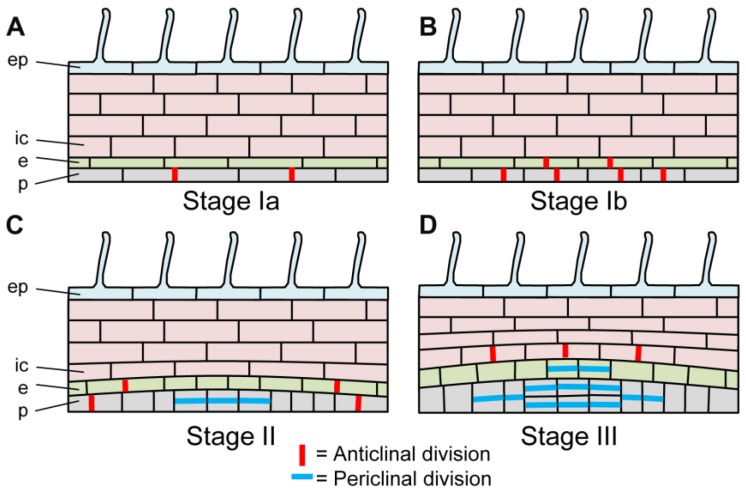
Schematic representation of the early developmental stages of lateral root formation in *Medicago truncatula*. Scheme of longitudinal sections in the main root of *M. truncatula* during LRF, showing the main type of cell divisions but not the precise number of dividing cells. (**A**). Stage Ia. Anticlinal divisions in the pericycle; (**B**). Stage Ib. Anticlinal divisions in the endodermis and the pericycle; (**C**). Stage II. Periclinal divisions in the pericycle and anticlinal divisions in the endodermis and the pericycle; (**D**). Stage III. Periclinal divisions in the endodermis (two cell layers) and the pericycle (four cell layers), and anticlinal divisions in the inner cortex. p: pericycle; e: endodermis; ic: inner cortex, ep: epidermis. From [61].

Indeed, unlike Arabidopsis but similarly to many other plants, LRF in *M. truncatula* does not only involve pericycle cell divisions but also a large contribution of the endodermis and innermost layers of the cortex (*cf.* Figure 3) [61,64]. Endodermal divisions were also described during white clover, arachis, and pea LRF [65,66,67,68], as well as in non-legumes [65,66,67]. Cortical cell divisions and contribution to the formation of the LR primordium (LRP) varies between species, even within legume species. For example, cortical cell divisions were shown to accompany LRF in Lotus but, in that case, a more important contribution of outer cortical cell layers was observed [64], reminiscent of the important contribution of the outer cortex layer for determinate nodule development; whereas inner cortical cell divisions are contributing more significantly to LRP formation in *M. truncatula*, that forms indeterminate nodules [61,64]. However, very little inner cortical cell divisions and contribution to the LRP was described in white clover [68]. Cortical cell divisions can contribute either to the formation of the LRP itself or facilitate LRP emergence [56]. Hence, endodermis and cortical contribution to the LRP are not specific to legumes but, interestingly, endodermal and cortical cell divisions are also involved in nodule formation [64,69,70].

Auxin perception zones have been extrapolated from DR5 or GH3 reporter systems both during LRF and nodule development in legumes. Hence, Mathesius *et al.* noticed a local increase in GH3:GUS expression in cortical cells after compatible rhizobium spot inoculation in white clover [71] and Pacios-Bras *et al.*, noticed GH3:GUS and GH3:GFP up-regulation more specifically in outer cortical cells after spot inoculation in *L. japonicus* reporter lines [72]. In the same way, Suzaki and co-workers observed a local high expression of DR5:GFP reporter upon nodule development in *L. japonicus* [60]. In the study of white clover, Mathesius *et al.* also noticed high GH3:GUS expression in dividing pericycle cells during LRF as well as strong GH3:GUS expression in cortical cells in front of developing LRP [71]. We have also noticed DR5:GUS expression in cortical cells above developing LRP in *M. truncatula* [61].

These evidences point at a role for auxin perception in nodule development as well as in preparing LR emergence in legumes, as observed in Arabidopsis [56].

As in Arabidopsis and many other plants, auxin plays the same major role for root development in legumes. We will briefly summarize some studies that have addressed the significance of auxin and particularly auxin perception and signaling, in LRF as well as in nodulation in legumes. Indeed, on top of its role in cell division and nodule development, auxin was also recently shown to be important for rhizobium infection as well [73].

### 3.1. Auxin Perception and Signaling

Auxin accumulation has been shown to be necessary for both LRF and nodule development. For example, application of auxin transport inhibitors such as NPA or TIBA results in pseudo-nodules formation in *Medicago* ssp. [6,74] and disruption of auxin transport by knocking down efflux transporter of the PIN family resulted in a reduced number of nodules in *M. truncatula* [75]. Moreover, a low concentration of auxin (<10^−7^ M of IAA) has been shown to enhance nodulation in *M. truncatula* [7]. Some rhizobium strains engineered to produce more auxin (Indole-3-Acetic Acid, IAA) can enhance nodule and LR development in *Medicago truncatula*—that forms indeterminate nodules—but has no effect on nodule number in *Phaseolus vulgaris*, which forms determinate nodules [8]. Turner and collaborators have down-regulated auxin perception by overexpressing the micro-RNA miR393 that induces mRNA degradation of auxin receptors, both in soybean and *M. truncatula* [76]. In both species, this down-regulation of auxin perception led to a decreased sensitivity to auxin for primary root elongation and LRF but opposite effects were observed on nodulation between these two species. Whereas miR393 over-expression resulted in less LR, it had no effect on nodulation in soybean [76], however, the same miR393 overexpression reduced both LR density and nodule number in *M. truncatula* [77]. This supports the hypothesis that determinate and indeterminate nodules do not have the same auxin requirement for their development (even if auxin perception is required for LRF in both species as shown by the reduced LR density observed in the miR393 overexpressing lines).

Other auxin signaling pathways components have been targeted using microRNA overexpression. Thus, overexpressing miR160, which targets the ARF10/16/17 family of auxin signaling repressors enhanced auxin sensitivity and drastically reduced nodule primordia development in soybean [76] and Medicago [78]. Another transcription factor (TF) that is regulated by auxin and miRNA is AtNAC1, that has been shown to positively regulate LRF in Arabidopsis [79]. Overexpression of miR164, that targets NAC1 reduced LRF but had no significant effect on nodulation in soybean [77] whereas it reduced nodule number without any effect on LRF in *M. truncatula* [80]. However, mutation in the *MtNAC1* gene was not sufficient to recapitulate this nodulation effect in Medicago [80]. This suggests again that auxin sensitivity required for determinate (as in soybean) and inderterminate (as in Medicago) nodule development is different.

Ta-siRNAs are also known to regulate auxin signaling pathway components such as the ARF3/4 transcription factors. Li *et al.* recently identified a mutant (*rel3*) involved in ta-siRNA production in *L. japonicus* and showed that this mutant displayed higher levels of ARF3 and ARF4 transcripts [81]. Interestingly, this mutant displayed a reduced primary root length and a reduced number of both LR and nodules. *Rel3* roots showed reduced auxin sensitivity and enhanced ethylene perception or synthesis since a normal nodule number could be restored by exogenous application of ethylene antagonists (AVG or Ag^+^) [81].

Altogether, this supports a positive role of auxin in nodulation and LRF in legumes.

An important cellular role of auxin is notably to control cell cycle progression (and hence cell divisions). Interestingly, Kuppusamy *et al.* identified a component of the APC/C degradation complex (that controls notably cyclins turnover), named *MtCDC16*, which is induced upon nodulation. *MtCDC16* is also expressed in root tips and LRP and is induced by auxin. *MtCDC16* knock-down using an RNAi construct reduces root sensitivity to auxin as well as root length and LRF but increases nodule numbers in Medicago [82].This points to a possible opposite control of some auxin downstream elements on nodulation and LRF in legumes. Whether this opposite effect is due to a control of the balance between LRP and nodule primordia or to something else need to be elucidated.

### 3.2. Auxin Transport and Biosynthesis

In addition to its signaling pathway, auxin can be regulated by its transport. The PIN family of auxin efflux carriers has for instance been shown to redirect auxin flow in different cell layers [58]. Huo *et al.* have silenced *PIN2*/*3*/*4* in *M. truncatula* and shown a negative effect on nodulation, although effect on LRF was not assessed [75]. Interestingly, some flavonoids, which are known to be produced by the host plant to establish nodulation, were also shown to act as auxin transport inhibitors [71]. Wasson *et al.* have silenced the flavonoid biosynthetic enzyme Chalcone Synthase (CHS) in *M. truncatula* and observed a decrease in nodule formation but no LRF default [83]. However, the same type of experiment (silencing of CHS) in soybean did not result in any major nodulation defects [84]. Once again, this points to differences between determinate and indeterminate nodules. The hypernodulation mutant *sunn* (*super numeric nodules*) was also shown to be affected in the long range shoot to root auxin transport modification induced upon rhizobial infection [7]. Indeed, *sunn* mutants did not show the reduction in auxin acropetal transport observed in wild type plants 24 h post rhizobial inoculation and this was hypothesized to be due to the lack of inhibition of auxin loading in the shoot following rhizobium inoculation in the *sunn* mutant [7]. This suggests that systemic control of nodulation [85] could also involve the regulation of shoot to root auxin loading and regulation of auxin concentration in the root. For a more complete view of the importance of auxin in nodulation see [86].

## 4. Endogenous Control of LRF by Other Hormones

Many other plant hormones control LRF, often in direct relation with auxin. Among these different hormones, we will review the major hormones for which root development data in legumes are available.

### 4.1. Cytokinins, Strigolactones, Gibberellic Acid, and Brassinosteroids

The main antagonists of auxin action are **cytokinins** (CK). These hormones are principally synthesized in the root but also in the aerial part of the plant [10]. CK have a promoting action on shoot branching but negatively regulate LRF, notably by negatively regulating auxin transport [10,87]. Held and collaborators have monitored CK response in both primary root and during nodule development in Lotus [88] using the synthetic TCS promoter [89]. In that way, they showed that CK response is high at the base of the LRP and later on in the root apex and transition zone. The TCS:GUS construct was also active throughout nodule development and in the Lotus root epidermis upon interaction with *Mesorhizobium loti* [88]. Loss of function in the cytokinin receptor genes *MtCRE1* and *LjLHK1* both reduces nodulation and enhances LRF [11,90], suggesting that the function of CK as negative regulators of LRF is conserved in legumes, where CK have evolved a positive role in nodule organogenesis [12]. Indeed, a gain of function mutation in the kinase domain of LjLHK1 results in spontaneous nodule development in the absence of rhizobium inoculation, pointing at the crucial role of CK perception in the onset of nodule development [91]. Downstream of CK perception are positive and negative regulators, named “response regulators” or RR genes. Type B-RRs are rapidly phosphorylated upon CK perception and regulate the transcription of type A-RRs that lack a DNA binding domain and are usually considered as negative regulators of CK signaling, as well as early and sensitive markers of CK signaling [10]. For example, Plet *et al.* have shown a rapid induction of the type A-RR *MtRR4* by CK, in a MtCRE1 dependent manner, and shown that *MtRR4* was expressed both during early stages of nodule development and later on in the apical zone of mature nodules. Interestingly, *MtCRE1* mutants show a more important acropetal auxin transport in their root tip, both under symbiotic (rhizobium inoculation) and non-symbiotic condition. This was accompanied by an increased expression of some *PIN* genes and an accumulation of PIN proteins in *Mtcre1* mutant roots [92]. Altogether, this suggests a negative feedback of CK signaling on auxin transport in *M. truncatula*, as observed in Arabidopsis. Lohar *et al.* have also shown, using an Arabidopsis promoter reporter fusion proARR5:GUS in *L. japonicus* roots, that this early target gene of CK signaling is not expressed in the pericycle undergoing cell divisions during early steps of LRF [93]. In the same way, down regulating CK levels of roots by overexpressing a cytokinin oxidase gene (that degrades CK) leads to an increase in LR and a decrease in nodule numbers in Lotus [93].

In an attempt to find nodulation specific RRs, Op den Camp *et al.* have looked at type A-RR genes that originated after the whole genome duplication that occurred in the Papilionoideae subfamily, with the hypothesis that these RRs could have been neo-functionalized to act during nodule development [64]. They found one pair of such genes, which they called *MtRR9* and *MtRR11* in *M. truncatula*. *MtRR9* and *MtRR11* expression was induced upon purified NF application, as well as during nodule development. However, knocking down the expression of *MtRR9* using an RNAi strategy reduced both LR and nodule numbers and over-expression of *MtRR9* led to the development of “nodule like” structure that were sharing both some of the nodule and LR development features, making it difficult to discriminate if *MtRR9* had any specific role in nodule development compared to LR development [64].

Thus, CKs do have a negative role on LRF in legumes and a positive role on nodulation (and especially on nodule organogenesis). How this dual regulation is achieved is not fully understood, although it may rely on a tissue specific action with a difference balance of auxin/cytokinins in pericycle and cortical tissues for instance.

**Strigolactones** (SGL) were identified as hormones that control shoot branching but also control root branching. Although the action of SGL seems dependent on phosphate concentrations in the growth medium, they seem to negatively regulate LRF in Arabidopsis, with a direct connection to auxin transport [35]. In *M. truncatula*, exogenous application of the SGL analog GR24 at 10^−7^ M reduced LRF and, although a transient stimulation of nodule number can be observed using 10^−7^ M GR24, application in the micromolar range strongly inhibited nodule development [36]. In contrast, Foo *et al.* reported that nodulation is reduced in SGL *Psccd7* and *Psccd8* biosynthesis mutants in pea (but enhanced in the *rms4* signaling mutant) [37,94]. The effect of GR24 on LRF in *M. truncatula* was observed on 19 day old plants in phosphate limiting conditions. Using the same growth conditions but younger (7 day old) plants, we observed a transient stimulation of LRF by 10^−7^ M GR24 application [95]. This may be linked to a different phosphate starvation status in younger plants that can benefit from cotyledon storage. However, a negative role for SGL in LRF in legumes is also suggested by the phenotype of the SGL biosynthesis mutants *Mtccd7* and *Mtccd8* [96]. Indeed, when grown under phosphate limiting conditions, both these mutants displayed a significantly higher number of LR than their corresponding wild type plants [97]. As found in Arabidopsis, SGL and auxin pathways are probably closely linked in legumes as well. Hence the semi-dominant mutant *bushy* (*bsh*) of pea shows a reduced auxin content (of approx 12-fold in the shoot and 3-fold in the root) and displays a root developmental phenotype and notably reduced LR root length and number ([98] or therein). Interestingly, the *bsh* mutant also shows reduced expression of the SGL biosynthetic gene *PsCCD8*, revealing a positive action of auxin on SGL biosynthesis [98]. Taken together, both genetic and pharmacological data suggests that SGL, in legumes as in Arabidopsis, are closely linked to auxin responses and that SGL are overall negative regulators of both LRF and nodulation in legumes, although their action may depend on the overall phosphate status of the plant.

**Gibberellic acid (GA) and Brassinosteroids (BR)** are phytohormones that control plant growth. Most of the legume mutants available so far in the GA and BR pathways were found in pea [31,99]. In pea, biosynthetic mutants of GA and BR form fewer LR and fewer nodules [31,99], although it may be difficult to quantify their effect exactly since they display a global dwarf phenotype. However, Maekawa *et al.* reported a negative role of GA on nodulation in *L. japonicus*, specifically, overexpression of a gain of function allele of *SLEEPY*, a F-box protein that acts as a positive regulator of GA signaling, resulted in reduced number of nodules but no overall effect on LR number in Lotus hairy roots [100]. Overall, the knowledge of the contribution of GA in the regulation of LRF and nodule development in legumes is still poor but should increase with the development of new genetic tools, and especially dominant negative regulators of GA signaling, as already done to test their role in mycorrhization [101].

For BR, Bazin *et al.* have shown that the miRNA miR396—that targets several growth-regulating factor genes (MtGRF) and two bHLH79—is induced by short BR treatment in *M. truncatula*. Overexpression of miR396 in *M. truncatula* affects primary root length and reduces arbuscular mycorrhizal colonization but not nodulation [102].

Other endogenous factors involved in LRF but with no obvious link to hormonal control have been documented in legumes. For instance, Boualem and collegues have shown that miR166 that targets class III HD-ZIP transcription factors (with known developmental roles in Arabidopsis) can control root and nodule development in *M. truncatula* [103]. Thus, overexpression of miR166 led to a decreased capability to form LR and nodules in hairy roots. Although miR166 has a link with auxin in Arabidopsis and despite a strong miR166 expression in LRP, nodule primordia and vascular tissues in *M. truncatula*, no link between the action of miR166 and auxin was shown in this study.

### 4.2. Stress Related Hormones

Although most plant hormones can be involved in stress responses, some of them have been often described as “stress related” and linked to abiotic (such as drought, salt…) or biotic (pathogen interaction) stresses.

One major hormone involved in abiotic (and biotic) stress and root development is **absicisic acid** (ABA) which has been shown to control both nodulation in several legumes [15,104] and, more recently, arbuscular mycorrhizal interactions in *M. truncatula* [105]. The ability of legumes to respond to ABA application, in a range of 10^−7^ to 10^−6^ M, by stimulating LRF and increasing LR density—instead of an inhibitory action in non-legumes and non-nodulating plants such as Arabidopsis—has been suggested to be a prerequisite for the ability to nodulate [5]. Interestingly, as summarized in Table 1, ABA has an antagonistic role in the regulation of LR and nodule development since the same concentration range that stimulates LRF actually inhibits nodulation [15]. This negative role of ABA was shown to be via an action on early NF signaling through inhibition of calcium spiking and, later on, by an action on nodule organogenesis through interaction with CK [15], and even on nodule senescence through the nitric oxide pathway [106]. During LRF, we have shown that 10^−7^ M ABA stimulates pre-emergence stages in *M. truncatula* [13]. Interestingly, ABA was also shown to rescue root meristem maintenance in the *latD* mutant of *M. truncatula*, mutated in a NRT1 member of the nitrate transporter family important both for nodule and root meristem maintenance [107,108]. These data point to another difference between legumes and Arabidopsis, in which ABA prevents LR emergence and does not promote meristematic activity [14]. However, this difference from Arabidopsis is lost at higher ABA concentrations. Indeed, Ariel *et al.* have shown that ABA was implicated in repressing LR emergence when applied in the 50 µM range [16].

Ariel *et al.* suggested that in *M. truncatula*, upon salt stress, ABA can induce the expression of the HD- ZIP transcription factor HB1 that, in turn, acts as a repressor of the LBD1 transcription factor that would mediates auxin induced LRF [16]. Another TF induced by **salt stress** and related to hormonal control is MtNAC969 [109]. Overexpression of this TF leads to less branched root systems while down regulation using an RNAi strategy leads to increased LRF. However, no effect on nodule number was observed [109]. Interestingly, MtNAC969 was rapidly induced by various hormone treatments such as CK, ABA and ethylene application in *M. truncatula* roots and by nitrate application in nodules. These data suggest that root development responses to salt stress could notably be mediated by ABA, through its action on the inhibition of LR emergence.

**Nitric Oxide** (NO) and ABA have been shown to cross-talk in many biological processes, including drought and other developmental responses [110], but the importance of NO in controlling root development in legumes is still poorly understood. Interestingly, NO accumulation was observed in LRP and nodule primordia in legumes [24]. Moreover, the increase in nodule number observed using a *S. meliloti* strain expressing the auxin biosynthesis genes *iaaM* and *tms2* was shown to be dependent on NO production since the NO scavenger cPTIO caused a significant decrease in nodule number of the Medicago plants inoculated with this strain [8]. Therefore, NO could be, as shown for other dicot species [22,23,25,26,27] a positive regulator of cell cycle reactivation, acting downstream of the auxin pathway in both LRP and nodule development in legumes.

**Ethylene** is another gaseous hormone involved in biotic stress and plant development. In legumes, ethylene is a negative regulator of nodulation since application of the ethylene precursor aminocyclopropane 1-carboxylic acid (ACC) prevents Nod Factor (NF) induced calcium spiking and nodule formation [111]. Moreover, the ethylene biosynthesis inhibitor aminoethoxyvinylglycine (AVG) or the ethylene receptor antagonist silver ion (Ag^+^) both enhance nodulation in several species [112,113]. In the same way, *Mtsickle*, a mutant in the *EIN2* gene, a component of the ethylene signaling pathway, shows a strongly enhanced number of nodules in Medicago as well as a better NF signaling [19,20,114]. The effect of the *Mtsickle* mutation on nodulation was shown to be related to a lack of auxin transport regulation triggered upon rhizobial inoculation [115]. For root developmental aspects, *Mtsickle* mutants show a more rapid primary root growth, which renders the measurement of LRF difficult to assess. However, studies in Lotus have shown that transgenic plants expressing the dominant negative form of the Arabidopsis ethylene receptor *etr1* were insensitive to ethylene and displayed fewer LR [21], a phenotype also found in the Arabidopsis *etr1-3* mutant [17], whereas the *Ljein2a* mutant “*enigma*” displayed higher ethylene content and a significant increase in LR number [112]. We have also addressed the effect of ACC on LRF in *M. truncatula* [116] and shown a dose dependent effect of ACC on LRF. Hence, concentrations up to 10^−7^ M ACC stimulated LRF of young *M. truncatula* seedlings whereas higher doses negatively regulated LR number. Altogether, this suggests that low ethylene concentrations stimulate LRF while high ethylene concentrations repress LRF. In Arabidopsis and tomato, 10^−6^ M ACC reduced both root elongation and LR number and 10^−7^ M ACC did not seem to positively affect LRF [17,18]. So, although high ethylene concentrations appear as a negative regulator of root elongation, LRF and nodulation in legumes, it may well be that sensitivity to low ethylene concentrations for LRF is slightly different between legume and non-legume plants but this requires further investigation.

The last “stress” related hormone we will discuss here is **jasmonic acid** (JA). This volatile molecule, and its biologically active derivative Methyl-Jasmonate (MeJA), is involved in pathogen interactions but has also been studied in a symbiotic context. Hence, Sun *et al.* have shown that JA inhibits NF induced calcium spiking and nodulation in *M. truncatula*, in a dose dependent manner and in a parallel and antagonistic manner to ethylene [29]. Sun *et al.* also noticed that, as for Arabidopsis, JA application (from 10^−7^ M) reduced primary root length of *M. truncatula*. Studies performed in soybean by over-expressing a MetJA biosynthesis gene, *NTR1*, showed that increased MetJA production resulted in an inhibition of nodulation and increased LR density [28]. These data suggest that JA has an antagonistic role on LRF and nodulation in legumes (*cf.* Table 1).

### 4.3. Small Regulatory Peptides

Peptides are also considered as hormones since they are endogenous products that can act at very low doses and at a distance from their site of production. In legumes, many of the regulatory peptides actions have been linked with nitrate—that is an important element controlling root development in all plants and nodulation in legumes—or with the negative systemic feedback loop known as “autoregulation of nodulation” that controls nodule numbers. We will review some of the effects of these small regulatory peptides relevant in legumes and their link with auxin when described.

Mutants impaired in the “autoregulation of nodulation” (AON) pathway often display root development phenotypes. For instance, different alleles of the *super numeric nodule* (*sunn*) mutant display reduced root length in non-symbiotic conditions [40]. The Lotus ortholog of *SUNN*, *HAR1*, shows a higher number of LR and a shorter primary root length in non-inoculated conditions [117], mutation in the orthologous gene of soybean, *nts1* also enhances LRF [42]. *SUNN* encodes a homolog of the Clavata1 LRR-RLK from Arabidopsis [40]. In Lotus, another LRR-RLK (unrelated to CLV1) named Klavier (KLV) was identified as being involved in AON [41]. Interestingly, a *klv* mutation is not additive to *har1* for nodulation indicating that both genes are involved in the same pathway for AON [118] but *klv* plants display shorter primary roots and a reduced number of LR in non-inoculated conditions [41]. In *M. truncatula*, Jin *et al.* found that 2.5 mM nitrate reduced LR density in wild type plants but not in *sunn* mutants. In this study, the authors also describe that LR density in the wild type plant is highly dependent on the nitrate content in the shoot and strongly correlated with shoot to root auxin transport. This link with nitrate content of the shoot and auxin transport is lost in the *sunn-1* mutant, suggesting that *SUNN* controls the LRF response to nitrate by integrating the nitrate content in the shoot and linking it with auxin shoot to root transport [119]. This is reminiscent of the shoot controlled regulation of AON by *sunn* [19]. LRR-RLKs usually have peptidic ligands. CLAVATA3/EMBRYO-SURROUNDING REGION (CLE) are small secreted peptides, some of which were shown to be CLV1 ligands. Some CLE peptides were also shown to be produced in the root and seem to mediate the AON signal from the site of nodulation in roots to systemic perception in shoots [120]. Recently, 35S:LjCLE-RS2 was shown to reduce LR number as well as nodule number in Lotus [121]. In *M. truncatula*, overexpression of CLE14 diminishes primary root length [122] but no effect on LRF was described. Interestingly a similar role for the CLE-CLV-1 pathway was recently demonstrated in systemic regulation of LRF in response to nitrate starvation in Arabidopsis [39]. Hence, overexpression of any of the four CLE peptides CLE 1, 3, 4 and 7 reduced LR growth and emergence, especially under high nitrate concentrations in Arabidopsis. This effect was lost in the *clv1* mutant [39]. Two of the closest homologs of these CLE peptides, named MtCLE12 and MtCLE13 are up-regulated in nodulated *M. truncatula* plants [122] and are closely related to LjCLE-RS1 and LjCLE-RS2. Interestingly, overexpression of MtCLE13 led to a strong reduction in nodule number (in a *SUNN* dependent manner) and MtCLE13 is regulated by CK [123]. 35S:MtCLE12 *in M. truncatula* inhibits nodulation, primary root growth and enhances GH3:GUS staining in the root, probably reflecting a change in auxin content/transport [124]. This suggests that the systematic regulation of nitrate status response through CLE-CLV1 signalling pathways is conserved in Arabidopsis and legumes, although CLE peptides are expressed in response to nitrate deprivation in Arabidopsis but in nitrate replete (during nodulation) conditions in legumes.

Some other peptides have been shown to mediate root developmental response to nitrate starvation. C-terminally Encoded Peptides (CEP) are small secreted peptides of 15 amino acids whose expression is induced by nitrate starvation (0 mM) or low (0.25 mM) nitrate concentration both in *M. truncatula* and in Arabidopsis [44,125]. Among the 11 CEP peptide genes found in Medicago, MtCEP1 was further characterized by Imin *et al.* [44]. MtCEP1 is expressed in root tips, vascular tissue, and young lateral organs and negatively regulates LRF but positively regulates nodulation. *MtCEP1* expression is not responsive to a 24 h treatment of 1 µM ABA/ACC/GA/GR24/BAP (synthetic CK)/NAA (synthetic auxin)/MeJA. External application of 1 µM of synthetic MtCEP1 significantly reduced LRF of several legume plants [44]. In contrast to the effect observed in Arabidopsis root development [43], MtCEP1 did not affect primary root elongation in *M. truncatula*. Further studies have shown that MtCEP1 activity depends on conserved post translational modifications such as hydroxylation of conserved proline residues. Hence the peptide encoded by the first coding region of MtCEP1 (D1) is predominantly hydroxylated at the Pro 4 and Pro 11 residues. In *M. truncatula* D1:HyP4,11 or D1:HyP4,7,11 have the most potent action on LRF at 0 mM nitrate. D2:HyP11 increased pre-emergence stages but not emergence of *M. truncatula* LR at 5 mM nitrate. This inhibition was not released by low (10^−10^ M) auxin external application [45]. NMR studies have shown that the conformational plasticity of CEP peptide is greatest when both P4 and P11 are post-translationally modified to hydroxy-proline residues [126].

Interestingly, two CEP receptors have recently been identified in Arabidopsis and were shown to be involved in a systemic response of root development to nitrate starvation in a shoot dependent manner [125]. Hence, CEP peptides applied to Arabidopsis roots can induce nitrate transporter expression in another part of the root, but only if the shoot of the plant is not mutated in the two CEP receptor genes. These receptors are two Leucine-Rich-Repeat Receptor Like Kinases (LRR-RLK), including *XIP1* that is involved in vasculature development in Arabidopsis [127]. Strikingly, the ortholog of *XIP1* in *M. truncatula* is the compact root architecture 2 (*CRA2*) gene, which is involved in systemic regulation of nodulation (in a nitrate independent pathway) and, antagonistically in the local regulation of LRF. *cra2* mutants display a strong increase in LRF-independent of the nitrate concentration of the growth medium- that is locally regulated in a root dependent manner; whereas *cra2* mutants display a strong reduction in nodule number dependent on the shoot genotype [128]. Hence the same LRR-RLK is involved in systemic regulation in *M. truncatula* and Arabidopsis, but has a role for nodule number regulation in Medicago and root developmental response to nitrate starvation in Arabidopsis. Interestingly, the antagonistic role of this pathway on LRF and nodule regulation in *M. truncatula* depends on two different (local *versus* systemic) regulation processes. Studies on the CEP peptides in *M. truncatula* have not addressed a systemic regulation effect yet. It is thus possible that CEP peptides have a local regulatory role on LRF and a potential systemic effect on nodulation in legumes.

Strikingly, the same peptidic (CLE and CEP) systemic regulatory pathways are conserved between Arabidopsis and legumes but have been directed toward LRF regulation by nitrate in Arabidopsis and regulation of nodulation in legumes.

Finally, a new family of small regulatory peptides, called miPEPs, has very recently been shown to regulate root development in both *M. truncatula* and Arabidopsis, although no possible links to nitrate or hormonal control have yet been studied [129].

## 5. Concluding Remarks

### 5.1. What Specificities for Legume LRF?

Legume root development, and especially LRF, is not fundamentally different from that of many plants from a cellular point of view. Even if endodermal and cortical contributions to the formation of LRP occur in several legumes [65,67,68] and if the ontogeny of nodules in legumes correlates with that of LR [64,69], this ability of endodermal and cortical cells to divide to form the LRP is not specific to legumes but is shared by many other plants [65,66,67]. In this respect, the pericycle limited origin of LRP in Arabidopsis appears to be the exception rather than the rule.

However, both determinate and indeterminate legume nodules do resemble LR, they originate from the same root cell layers and have their development controlled by phytohormones.

### 5.2. Dual Hormonal Control of LRF/Nodulation in Legumes

Interestingly, some hormones do have the same action on LR and nodulation while others seem to have evolved antagonistic action on these two lateral organs. For example, auxin, although maybe not with the same level of sensitivity, is a positive regulator of both LR and nodule development and its accumulation and transport is crucial for the onset of these two developmental programs. Other hormones, like ABA, CK, JA, and ethylene play opposite roles between LRF and nodulation in legumes. ABA, JA, and ethylene are positive regulators of LRF and negative regulators of nodulation while CK are negative regulators of LRF and positive regulators of nodulation.

### 5.3. Comparison with LRF Hormonal Control in Other Dicots

Auxin, CK, JA, and strigolactones seem to play similar roles in controlling LRF in legumes and other dicots such as Arabidopsis or tomato (see Table 1). However, low doses of ABA and ethylene stimulate LRF in legumes, but not in non-nodulating plants for ABA and at least not in Arabidopsis or tomato for ethylene [5,17,18]. It is thus possible, as already suggested by Liang and colleagues for ABA [5], that different hormonal sensitivities have been a pre-requisite for plants to develop nodulation, maybe thanks to this capability to display antagonistic control of two different lateral organs with the same hormones. More extensive studies on nodulating vs non-nodulating plants should be conducted to clarify if root development sensitivity to other hormones than ABA could account for a general feature of nodulating *vs.* non-nodulating plants.

### 5.4. Future Directions

Interestingly, non-legume actinorhizal plants (that form a symbiotic interaction with the nitrogen fixing bacteria *Frankia*) and *Parasponia* (the only non-legume plant to establish a root endosymbiosis with rhizobia) do form a different type of nodule, much more developmentally related to LR. For instance, actinorhizal and *Parasponia* nodules originate from the pericycle and have a central vasculature, as for LR, whereas legume nodules have a peripheral vasculature ([130] for review). Molecular comparison of LR and nodule developmental programs in legumes, actinorhizal plants and *Parasponia* could be an interesting way to understand the specificities and overlaps of these developmental pathways.

Another issue that needs clarification is the possible balance that exists between LR and nodule numbers. So far, contradictory evidence exists and it is not clear if increasing nodule number reduces LRF or vice versa. The fact that some components of the auxin pathway (like *MtCDC16* in Medicago) can play antagonistic roles in LRF and nodule development or that the level of auxin sensitivity required for LRF and determinate nodule development seems slightly different, for example, opens a way towards understanding how a difference in auxin sensitivity or signaling could possibly balance LR and nodule numbers.

In contrast to Arabidopsis, and apart from the extensive SGL pathway mutant collection in pea, little genetic dissection of the hormonal pathways has been performed in legumes, mainly because of the lack of extensive appropriate mutant collections. For instance, auxin perception/signaling/transport/biosynthesis mutants are mostly still missing. With the completion of many legume genomes and the development of mutant libraries, this gap is starting to be filled slowly and systematic studies of hormonal pathways are emerging [131,132]. Although these recent studies are still mainly focused on symbiotic interactions such as nodulation or mycorrhization, this focus will provide new tools to better address and dissect the roles of these signaling pathways on root development as well. Moreover, a systematic approach to address the effects of these hormones, alone or in interaction, in both root development and symbiotic responses remains a real challenge that needs to be addressed in order to better understand the regulatory complexity of these different developmental outcomes occurring in legume roots.
